# Implant periapical lesion: Diagnosis and treatment

**DOI:** 10.4317/medoral.17996

**Published:** 2012-08-28

**Authors:** María Peñarrocha-Diago, Laura Maestre-Ferrín, Juan Cervera-Ballester, David Peñarrocha-Oltra

**Affiliations:** 1 Associate Professor of Oral Surgery. Master of Oral Surgery and Implantology. Valencia University Medical and Dental School, Valencia, Spain; 2 Master of Oral Surgery and Implantology. Valencia University Medical and Dental School; 3 Student of Master of Oral Surgery and Implantology. Valencia University Medical and Dental School, Valencia, Spain

## Abstract

The implant periapical lesion is the infectious-inflammatory process of the tissues surrounding the implant apex. It may be caused by different factors: contamination of the implant surface, overheating of bone during drilling, preparation of a longer implant bed than the implant itself, and pre-existing bone disease. Diagnosis is achieved by studying the presence of symptoms and signs such us pain, swelling, suppuration or fistula; in the radiograph an implant periapical radiolucency may appear. A diagnostic classification is proposed to establish the stage of the lesion, and determine the best treatment option accordingly. The following stages are distinguished: acute apical periimplantitis (non-suppurated and suppurated) and subcacute (or suppurated-fistulized) apical periimplantitis. The most adequate treatment of this pathology in the acute stage and in the subacute stage if there is no loss of implant stability is apical surgery. In the subacute stage, if there is implant mobility, the extraction of the implant is necessary.

** Key words:**Implant periapical lesion, apical periimplantitis, retrograde periimplantitis.

## Introduction

The increasing popularity of implants has led to a considerable increase in the incidence of implant periapical lesions (1). This pathology, unless diagnosed and treated early, may lead to implant failure in the first weeks after its placement.

Palma-Carrió et al. (2) conducted a literature review about risk factors associated to early failure of dental implants. They found that early failure rates (those occurred before implant loading) vary from 1.2 to 3% and late failure (after implant loading) rates from 0 to 1.8%. According to studies included in their review, statistically significant factors associated with early implant failure were smoking, poor bone quantity and quality, and implant location in posterior regions; however few studies specified the related risk factors.

Implant periapical lesion may often be the cause of early failures, as this pathology is difficult to diagnose and it has been rarely studied in the literature. We propose a diagnostic classification to help identify this disease in its early stages, and establish the appropriate treatment accordingly.

## Concept

Implant periapical lesion, also referred to as apical periimplantitis or retrograde periimplantitis, was first described by McAllister in 1992 (3). Sussman and Moss (4) defined it as the infectious-inflammatory process of the tissues surrounding the implant apex; and Quirynen et al. (5) as a clinically symptomatic periapical lesion that develops shortly after implant insertion while the coronal portion of the implant achieves a normal bone to implant interface.

## Prevalence

The prevalence of this pathology is low. Reiser and Nevins (6) found 10 cases in 3800 implants placed (0.26 %); Quirynen et al. (5), in a retrospective study involving 539 implants, obtained a prevalence of 1.6 % in the maxilla and 2.7 % in the mandible, diagnosing all cases before the second stage surgery.

## Etiology

Among the factors related with the apparition of this pathology are: contamination of the implant surface (7,8), overheating of bone during drilling (7,9), preparation of a longer implant bed than the implant itself (6), preexisting bone disease (10), presence of residual root fragments or foreign bodies (6,7) and implant placement in proximity to an infected maxillary sinus (11).

For some authors the most likely cause is endodontic pathology of the tooth replaced by the implant or the adjacent tooth (5,12). Ayangco and Sheridan (10) published three cases of implant periapical lesions in patients in whom failure of apical surgery of the teeth had occurred before implant placement. According to the authors, despite the curettage of the sockets and the prolonged waiting time until implant insertion, bacteria could remain in the bone causing subsequent development of lesions in the implants. Meanwhile, Brisman et al. (13) associated the failure of four implants to the existence of adjacent endodontically treated teeth, which were asymptomatic and showed no radiographic signs of pathology. Sussman (14) classified the lesions into: implant to tooth (type I), when the neighboring tooth is injured during implant drilling, and tooth to implant (type II), when the lesion occurs due to contamination of the implant from an apical lesion of the adjacent tooth. Balshi et al. (15), suggested that the etiology of this process is multifactorial and was unable to confirm or reject any of the above hypothesis.

## Diagnosis

Diagnosis of implant periapical lesions is clinic and radiographic. Symptoms and clinical signs which may appear are pain, swelling, suppuration and fistula; in the radiograph an implant periapical radiolucency may be identified in some cases. Reiser and Nevins (6) classified implant periapical lesions into inactive (or not infected) and active (or infected).

The inactive form is asymptomatic and it is diagnosed because of the presence of a radiolucency around the apex of the implant. This radiolucency is an apical scar caused by vertical overpreparation of the implant bed or by bone necroses due to overheating during implant insertion. Inactive lesions do not require treatment unless the radiolucency grows in size; these lesions should be controlled radiographically.

In the active form, the lesion is symptomatic and requires treatment to avoid the progress of bone destruction. Along with periapical radiolucency other signs and symptoms may appear: gingival reddening, painful soft swelled mucosa and, in some cases, presence of a fistulous tract.

The diagnosis must include determination of the evolution stage of the lesion in order to apply the best treatment option. In the non-suppurated acute apical periimplantitis, there is an acute inflammatory infiltrate, and it is clinically characterized by the presence of acute spontaneous and localized pain, which does not increase with percussion; the mucosa can be swelled and painful and implant percussion produces a tympanic sound; in the radiograph no changes in bone density can be seen around the implant apex. Progression leads to a suppurated acute apical periimplantits or apical abcess, where a purulent collection is formed around the implant apex. Clinic is similar to that of the non-supurated stage, but an implant periapical radiolucency is observed.  Table 1 summarizes signs and symptoms of each stage and compares them with those taking place in teeth.

**Table 1:**
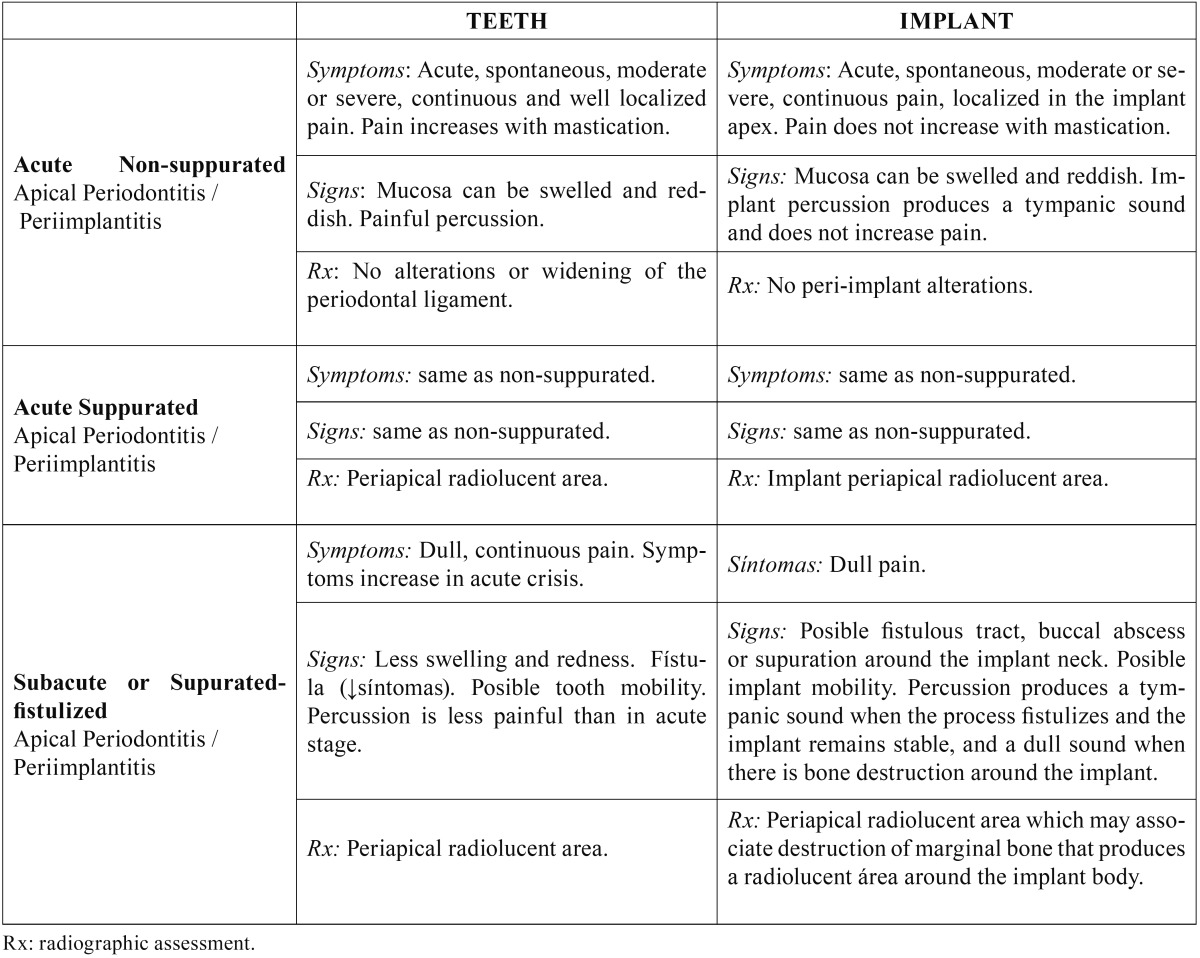
Stages in the evolution of apical periodontitis-periimplantitis.

The purulent collection, typical of the suppurated stage, looks for least resistance drainage pathways and destroys bone around the implant; once the drainage pathways is created a subacute apical periimplantitis (or suppurated-fistulized apical periimplantitis) is established. If the coronal bone-implant junction is well consolidated a fistulous tract develops from the implant ápex to the buccal cortical; a buccal abscess may thus arouse. On the contrary, if the coronal bone-implant junction is not well consolidated, this will be the least resistant drainage pathway; peri-implant bone will be destroyed coronally and the implant will be lost as a result. In this subacute stage the symptoms are not marked; there may be a fistulous tract, a buccal abscess or suppuration around the implant neck. Depending on the progress of the process the implant may be mobile and, in the radiograph, bone destruction along the body of the implant may be seen.

## Treatment

The correct diagnosis of these lesions in their early stages allows their early treatment, and prevents implant failure. In a patient presenting with acute pain, well localized in relation to the apex of the implant, after a short period (1 to 3 weeks) since implant placement, presence of an acute apical periimplantitis (suppurated or non-suppurated, depending on the existence of apical radiolucency or not) must be suspected and surgical treatment should be performed (implant apical surgery).

Periapical radiolucencies may sometimes be casual findings during routine radiographic assessments. If the patient is asymptomatic and the diameter of the radiolucent area is small, it is not necessary to treat the lesion; overpreparation of the implant bed is the most probable cause, and only periodic radiographic controls should be done. If, in any of the controls, the radiolucency has increased in size or the patient develops symptoms, the surgical treatment will be applied.

**Figure 1:**
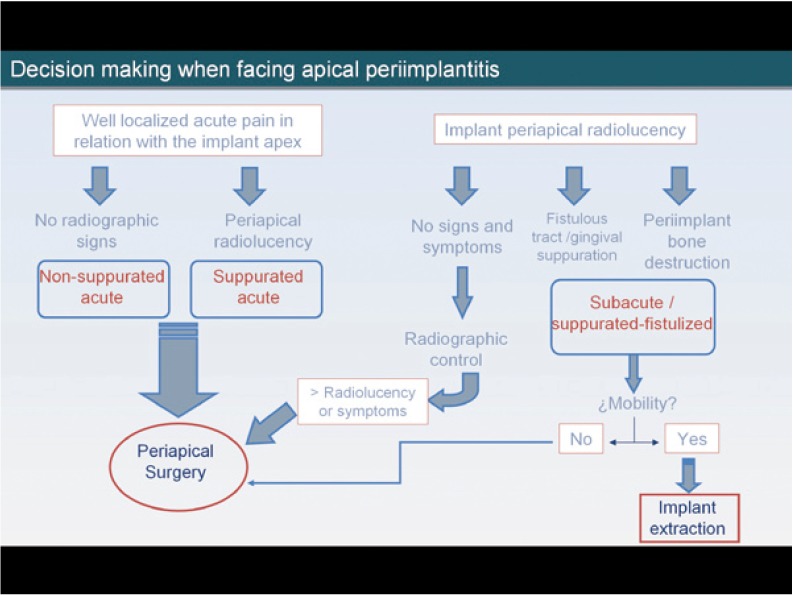
Decision making when facing apical periimplantitis.

In the subacute stage, the symptoms are less marked but bone destruction is greater. The apical radiolucent area may be accompanied by bone destruction around the implant body, and soft tissue signs such as a fistulous tract or a vestibular abscess may be present. In these cases we must ensure that the stability of the implant has not been damaged; if the implant is mobile, it must be extracted, and if not, implant apical surgery must be performed (Fig. 1).

The most studied treatment of implant periapical lesions with no associated implant mobility is implant apical surgery. Most authors curettaged the lesion and irrigated with saline solution (5,10,16). Several agents have been applied for decontamination of the implant surface, such as chlorhexidine (16-18) or tetracycline pastes (10,15,18), but there is no evidence of the efficiency of any of them. Sometimes, bone regeneration materials are used, accompanied or not with tissue regeneration barriers, in order to achieve complete bone regeneration of the defect (5,19,20). Other authors (10,15,21), suggest sectioning the implant apex in those cases in which total removal of the granular tissue is not assured, and when working within the maxillary sinus or nasal cavity. Scarano et al. (11) decided to remove the implant in a patient because of pain persistence after treatment with analgesics; Oh et al. (22) removed one implant which presented mobility, and Sussman (14) recommended to remove the implant in all cases to prevent the onset of osteomyelitis. On the other hand, Tözüm et al. (20) and Zhou et al. (12) performed root canal retreatment or periapical surgery if the adjacent tooth was endodontically treated.

Waasdrop and Reynolds (23) suggested that asymptomatic implant periapical lesions could be resolved by antibiotic therapy without surgical intervention, as a lesion of this type was fully resolved after treatment with antibiotics (amoxicillin 500 mg three times a day during 10 days). According to these authors the apical radiolucency presented by their patient was caused by an overpreparation of the implant bed, and the patient never showed signs of infection or inflammation; thus the diagnosis of apical periimplantitis is uncertain. Other authors consider that in the presence of inactive or asymptomatic lesions treatment is not indi-cated (6). According to some published case series (16,21), initial treatment with antibiotics was not effective to control sympto-matic or active lesions, which required surgical access. Romanos et al. (24) concluded in their review that antibiotic treatment alone is not effective.

## Prognosis

Romanos et al. (24) studied the prognosis of implant apical lesions after reviewing all cases published up to December 2007; 75% of the implants diagnosed with periapical lesion survived after treatment, with follow-up periods ranging between 4 months and 7 years. Most studies reported few clinical cases, and it is difficult to determine the prognosis of implants treated with periapical surgery.

## Conclusion

Apical peri-implantitis is classified according to evolution stages into acute (non-suppurated and suppurated) and subacute (or suppurated-fistulized). In the acute stage and in the subacute if there is no loss of implant stability, the correct treatment approach is implant periapical surgery. In the subacute stage associated with implant mobility the implant must be removed.
